# Gli1 maintains cell survival by up-regulating IGFBP6 and Bcl-2 through promoter regions in parallel manner in pancreatic cancer cells

**DOI:** 10.4103/1477-3163.55429

**Published:** 2009-09-03

**Authors:** Xuan-Fu Xu, Chuan-Yong Guo, Jun Liu, Wen-Juan Yang, Yu-Jing Xia, Ling Xu, Yong-Chun Yu, Xing-Peng Wang

**Affiliations:** Department of Gastroenterology, The Tenth Hospital, Tongji University, Shanghai, China; 1 Xuan-Fu Xu and Chuan-Yong Guo contributed equally to this paper

**Keywords:** Pancreatic Neoplasm, Hedgehog Signaling Pathway, Gli1, IGFBP6, Bcl-2, Apoptosis

## Abstract

**Background:**

Aberrant activation of Hedgehog (Hh) signaling pathway has been reported to be related to malignant biological behavior of pancreatic cancer but its mechanism is unclear yet. Since IGF pathway and Bcl-2 family are involved in proliferation and apoptosis of pancreatic cancer cells, we hypothesize that they are possibly associated with Hh pathway.

**Materials and Methods:**

We studied the relationship of Shh-Gli1 signaling pathway with proliferation and apoptosis of pancreatic cancer cells and the regulation of transcription factor Gli1 to insulin-like growth factor binding protein 6 (IGFBP6) and Bcl-2 genes at the level of transcription.

**Results:**

Sonic hedgehog (Shh), Smoothened (Smo), patched and Gli1 were expressed in pancreatic cancer cells. Cyclopamine inhibited cell proliferation at low concentration and induced apoptosis at high concentration. Effect of RNA interference (RNAi) for Gli1 to cell survival is mainly due to proliferation inhibition though involved in apoptosis. The transcription factor Gli1 bound to promoter regions of Bcl-2 and IGFBP6 genes and the levels of IGFBP6, proliferating cell nuclear antigen (PCNA) and Bcl-2 messenger RNA (mRNA) were decreased as well as Gli1 mRNA significantly by cyclopamine or RNAi in cultured pancreatic cancer cells (p < 0.01). Finally PCNA, IGFBP6 and Bcl-2 mRNA were upregulated as well as Shh or Gli1 in pancreatic cancer tissues (p < 0.01).

**Conclusions:**

Our study reveals that Gli1 maintained cell survival by binding the promoter regions and facilitating transcription of IGFBP6 and Bcl-2 genes in a parallel manner in pancreatic cancer cells and suggests it may be one of the mechanisms of Shh-Gli1 signaling pathway in pancreatic cancer.

## INTRODUCTION

Pancreatic cancer is the fourth or fifth leading cause of death from cancers in many different countries.[1,2] Because the pancreatic cancer is characterized by extensive local invasion and early lymphatic and hematogenous metastasis, only 10 to 20% of patients are suitable for surgical resection, contributing to an overall 5 - year survival-rate of less than 5%.[3]

The reasons for poor prognosis of pancreatic cancer are mainly due to different gene mutations. So far, the proposed model of molecular mechanisms of pancreatic cancer consists of mutation and overexpression of some oncogenes such as K-ras and HER-2/neu followed by inactivation of some tumor suppressor genes such as p16, p53, DPC4 and BRCA2 that lead to a series of aberrant gene expression or signaling pathway activation involved in cell-cycle, apoptosis, angiogenesis, metastasis and differentiation that alter cell survival, increasing the aggressiveness and conferring resistance to conventional chemotherapy and radiotherapy.[4] Therefore, it is very important to search new molecules that selectively inhibit these mutations or aberrant signaling pathways from the mutations.

Recently, hedgehog (Hh) signaling pathway has been proven to be an important promoter of tumor growth in various cancer entities, and it has been considered a target for therapy.[5] Hedgehog signaling is involved in the development of the pancreas. Inappropriate activation of Hh signaling during pancreas formation results in several diseases and agenesis of this organ.[6] Aberrant activation of Hh pathway resulting from the upregulation of sonic hedgehog (Shh) gene has been found in the majority of human pancreatic cancers.[7] Moreover, blocking Hh signaling inhibited tumor growth, angiogenesis and metastasis significantly.[8,9] Interestingly, Hh is excluded from the developing pancreas, as well as the mature organ, but activated in early PanIN lesions and invasive pancreatic cancer.[10] Thus, the evidence suggests that activated Hh signaling is a critical early mediator of pancreatic cancer development. Transcription factors of Hh signaling cascade, glioma-associated antigen (Gli), have three homologs, Gli1, Gli2 and Gli3, in mammals which share a highly conserved zinc-finger domain and are believed to function as the most downstream components in the vertebrate.[11,12] It has been proposed, based on identification of Gli1 direct target genes, that Gli1 genes are important to cell proliferation, apoptosis, cell adhesion, metastatic potential and angiogenesis.[13–15]

There is a large body of evidence showing that IGF pathway and Bcl-2 family are involved in the regulation of cell proliferation and apoptosis in pancreatic cancer. Since IGFBP6 and Bcl-2 genes have been proved to be target genes of transcription factor Gli1, we hypothesize that Shh-Gli1 pathway are associated with IGF pathway and Bcl-2 family in pancreatic cancer. As IGFBP6 binds IGF2 and Bcl-2 binds Bax or Bak1 usually *in vivo*, we can speculate that Shh-Gli1 signaling may maintain cell survival through building some balance mechanisms between Bcl-2 and Bak1 or Bax as well as between IGFBP6 and IGF2 by mediating transcription of these genes.[16,17]

## MATERIALS AND METHODS

### Human Pancreatic Cancer Specimens

Pancreatic cancer and corresponding cancer side tissues used for RNA extraction were obtained from six patients undergoing resection for pancreatic cancer at the Tenth Hospital of Tongji University, Shanghai, China. Tissue samples were frozen in liquid nitrogen at the Pancreatic Disease Key Laboratory of Shanghai, China. All patients have signed a consent document, in which they agreed before their operations that the surgical excision of malignancy would be used in medical research. The human subject committees of the Tongji Universities approved the studies.

### Cell cultures

Human pancreatic cancer cell lines were grown in medium (RPMI1640 supplemented with 2% FBS for BxPC-3 and DMEM with 5% FBS for AsPC-1, Panc-1 and SW1990) at 37°C in 5% CO_2_ and 95% air-humidified incubator. Cyclopamine (Merck, Darmstadt, Germany) was dissolved in 100% DMSO and then diluted fresh on the day of testing for cell culture experiments. The final concentration of DMSO for all treatments (including controls, where no drug was added) was maintained at 0.1%.

### RT-PCR

Total RNA was extracted from BxPC-3, AsPC-1, Panc-1 and SW1990 cells by using Trizol reagent (Invitrogen, California, USA) and total RNA (2*μ*g) was reverse-transcribed with reverse transcriptase (Promega, Madison, USA) according to the manufacturer's instructions. All primers were obtained from Generay Biotech (Shanghai, China). The primer sequences were 5'-CGGAGCGAGGAAGGGAAAG-3' (sense) and 5'-TTGGGGATAAACTGCTTGTAGGC-3' (antisense) for Shh (262bp), 5'-CGGCGTTCTCAATGGGCTGGTTTT-3' (sense) and 5'-GTGGGGCTGCTGTTTCGGGTTCG-3' (antisense) for Patched (376 bp), 5'-ACCCCGGGCTGCTGAGTGAGAAG-3' (sense) and 5'-TGGGCCCAGGCAGAGGAGACATC3' (antisense) for Smoothened (Smo) (562bp), 5'-TCTGCCCCCATTGCCCACTTG-3' (sense) and 5'-TACATAGCCCCCAGCCCATACCTC-3' (antisense) for Gli1 (480 bp), 5'-GCATCGTGATGGCTCCG-3' (sense) and 5'-GCTGGAAGGTGGACAGCGA-3' (antisense) for β-actin (125 bp). After 30 cycles at the annealing temperatures, 54°C for β-actin, Shh, Patched, Gli1 and 58°C for Smo, the products of PCR were visualized by 1.5% agarose gel.

### RNA interference (RNAi)

RNA oligonucleotides of RNAi for Gli1 (5' - CCACAGCACCATGACTAGT-3'/5'- GGTGTCGTGGTACTGATCA-3') were synthesized by Geneman Biotech (Shanghai, China). Sense and antisense oligonucleotides were resuspended to 50 *μ*mol/L in diethyl pyrocarbonate-treated water, mixed, heated for 2 min at 90°C and annealed for 1 h at 37°C. Annealed oligos (20 *μ*mol/L) were aliquoted and stored at −80°C. For RNAi, pancreatic cancer cell lines were incubated overnight, washed, incubated in the serum-free medium and exposed to duplexed siRNA oligonucleotides in the presence of Oligofectamine (Invitrogen, California, USA) for 6 h and then the media was changed to medium with FBS.

### Cell Proliferation assay

Cell proliferation was determined by the MTT [3-(4, 5 dimethyl-2-thiazolyl)-2.5-diphenyl- 2H-tetrazolium bromide] assay as described elsewhere.[20] Briefly, human pancreatic cancer cell lines were plated in a 96-well plate. After 12 h of incubation in serum-free medium, the pancreatic cancer cells were cultured in corresponding medium containing 5, 10, 15 or 20 *μ*M of cyclopamine, respectively. Purmorphamine (Cayman chemical, Michigan, USA) at different concentrations (5, 10, 15 and 20 *μ*M) was used to counteract efficacy of cyclopamine to pancreatic cancer cells treated with 15 *μ*M cyclopamine. For RNAi, human pancreatic cancer cells were exposed to 25, 50, 100 or 200 nM of duplexed siRNA oligonucleotides, respectively. After four days, MTT assay was carried out and optical density was read with an ELISA reader at a wavelength of 490 nm.

### Annexin V-FITC/propidium iodide (PI) assay

Annexin V-FITC kit (BD Pharmingen, San Jose, CA) was used according to the manufacturer's protocol to detect phosphatidylserine translocation from the inner to the outer plasma membrane, an early marker of apoptotic cell death. Briefly, after pancreatic cancer cells were plated in 10 cm dishes and placed on coverslips, the cells were treated with 50, 100, 150 and 200 *μ*M cyclopamine for 24 h and 15 *μ*M purmorphamine was used to counteract efficacy of cyclopamine to pancreatic cancer cells as a control. For RNAi, pancreatic cancer cells were exposed to 25, 50, 100 and 200 nM duplexed siRNA oligonucleotides for 6 h and then grown for 24 h. Cells on coverslips were incubated with annexin V-FITC (1 *μ*g/mL, final concentration) and PI (40 *μ*g/mL, final concentration) at room temperature for 15 min in the dark and observed under a fluorescence microscope. Cells in the dishes were collected and labeled similarly and acquired on flow cytometer immediately.

### DNA fragmentation assay

Pancreatic cancer cell lines BxPC-3, AsPC-1, Panc-1 and SW1990 were treated with 150 *μ*M cyclopamine or 100 nM siRNA for Gli1 for 24 h. DNA fragmentation assay was performed as previously. In brief, cells were washed twice with PBS and lysed by addition of a hypotonic solution (1% NP-40 in 20 mM EDTA, 50 mM Tris-HCl pH 7.5). The supernatants were treated with RNase A and proteinase K. Before hydrolysis, a further purification of DNA was performed by phenol-chloroform extraction, followed by three successive ethanol precipitations in 2 M ammonium acetate. Pellets were dried and resuspended in 200 *μ*L TE. Aliquots containing 20 *μ*g DNA were electrophoresed in 2% agarose gel.

### Formaldehyde Cross-linking Chromatin Immunoprecipitation (XChIP)

XChIP was carried out as the previous studies.[18,19] In brief, cells were treated with formaldehyde at a final concentration of 0.8% for 10 min at room temperature and then cross-linking reactions were terminated by adding glycine to a final concentration of 0.125 M. Cells from one dish (approximately 2 × 10^7^ cells total) were lysed with 1.0 mL IP buffer containing the protease inhibitor cocktails. Chromatin was sheared by using a sonicator with a 4 mm tip probe for 6 times of 10 s pulses (350 W, 60 s intervals) in a -20°C ice box. Cross-linking was reversed by adding 20 *μ*L of 5 M NaCl overnight at 65°C and DNA was extracted using phenol/chloroform assay. A 20 *μ*L of DNA was electrophoresed in 2% agarose gel and the rest was saved in -20°C as a positive control template for PCR.

The chromatin solution were precleared by adding 50 *μ*L normal rabbit serum for 30 min at 4°C, incubated with protein A/G PLUS agarose, rotated for 40 min at 4°C and centrifuged at full speed for 1 min at 4°C. The supernatants were transferred to fresh tubes. ChIP-grade goat polyclonal Gli1 antibody (Santa Cruz Biotechnology, California, USA) (0.1 *μ*g/mL) was added to the supernatant overnight at 4°C and the mouse IgG (Santa Cruz Biotechnology, California, USA) (0.1 *μ*g/mL) was added as negative control and then 50 *μ*L Protein A/G PLUS-Agarose (Santa Cruz Biotechnology, California, USA) was put into and the slurry was rotated for 1 h at 4°C. And then the beads were washed five times with 1 mL cold IP buffer containing inhibitors and two times with TE buffer. DNA was eluted twice from the Protein A/G PLUS-agarose beads with 250 *μ*L of elution buffer (1% SDS and 0.1 M NaHCO_3_). Cross-linking was reversed and DNA was extracted as described above. Samples were analyzed by PCR. The primers designed from the promoters of IGFBP6, Bcl-2 and GAPDH were 5'-CGGGAACAAAGCAAGAAA-3' (sense) and 5'-CTAAACTGGGTGGGACTGG-3' (antisense) for IGFBP6 (122bp), 5'-GCAGGCCTGAGCAGAAGG-3' (sense) and 5'-AACGTCACACGGTTCATTCA-3' (antisense) for Bcl-2 (300 bp), 5'-TACTAGCGGTTTTACGGGCG-3' (sense) and 5'-TCGAACAGGAGGAGCAGAGAGCGA-3' (antisense) for GAPDH (166 bp). After 32 cycles at the annealing temperature, 54°C for IGFBP6 and 59°C for Bcl-2 or GAPDH, PCR products were analyzed by 1.5% agarose gel.

### Immunoprecipitation Western-blotting (IP-western)

To detect expression of Gli1 protein in pancreatic cancer cells and to validate the specificity of the Gli1 antibody used in XChIP assay, IP-western assay was carried out under the same condition with XChIP-PCR assay. ChIP-grade goat polyclonal Gli1 antibody was used as above, mouse monoclonal β-actin antibody (Santa Cruz Biotechnology, California, USA) (0.1 *μ*g/mL) was used as internal reference and the mouse IgG (Santa Cruz Biotechnology, California, USA) (0.1 *μ*g/mL) was added as negative control. After final wash the beads were resuspended in sample buffer (250 mM Tris–HCl, 4% SDS, 10% glycerol, 0.006% bromophenol blue and 2% β-mercaptoethanol), boiled for 5 min, cooled on ice for 5 min and size-fractionated on 6% SDS–PAGE gels for Gli1 protein or 12% SDS–PAGE gels for β-actin. Proteins were transferred onto PVDF membranes at 300 mA for 3 h for Gli1 protein and 150 mA for 1 h for β-actin protein. Subsequently, membranes were incubated for 1 h in a blocking solution (5% non-fat milk in 20 mM Tris–HCl, 150 mM NaCl, 0.1% Tween-20), followed by incubation with rabbit polyclonal Gli1 antibody (Santa Cruz Biotechnology, California, USA) or mouse monoclonal β-actin antibody (Santa Cruz Biotechnology) in blocking solution overnight at 4°C. Protein was visualized by chemoluminescence western blot detection system (Boster Company, Wuhan, China).

### Quantitative real-time RT-PCR

Total RNA was extracted from pancreatic cancer and corresponding cancer side tissues with Trizol reagent (Invitrogen, California, USA) according to the manufacturer's protocol. After being treated with 15 *μ*M cyclopamine or exposed to 100 nM RNAi oligonucleotide for 24 h the total RNA of p ancreatic cancer cell lines was extracted as above. QRT–PCR was performed with the Light Cycler Fast Start DNA SYBR Green kit (TaKaRa Biotechnology, Dalian, China). Total RNA (100 ng) was reverse transcribed in 25 *μ*L volume using the first strand cDNA synthesis kit for RT-PCR according to the manufacturer's instructions. All primers were obtained from Generay Biotech (Shanghai, China). The primer sequences, annealing temperatures and amplification sizes for each of the genes were as follows: 5'-TCTGCCCCCATTGCCCACTTG-3' (sense) and 5'-TACATAGCCCCCAGCCCATACCTC-3' (antisense) for Gli1 (54°C, 480bp), 5'-CGGAGCGAGGAAGGGAAAG-3' (sense) and 5'-TTGGGGATAAACTGCTTGTAGGC-3' (antisense) for Shh (54°C, 262bp), 5'-GCCGAGATCTCAGCCATAATT-3' (sense) and 5'-ATGTACTTAGAGGTACAAAT-3' (antisense) for PCNA (55°C, 453bp), 5'-AATCCAGGCACCTCTACCA-3' (sense) and 5'-GCCCATCCGATCCACA-3' (antisense) for IGFBP6 (50°C, 240bp), 5'-GCATTGCTGCTTACCG-3' (sense) and 5'-TTTGCTCACTTCCGATT-3' (antisense) for IGF2 (50°C, 484bp), 5'-GTGAACTGGGGGAGGATTGT-3' (sense) and 5'-GGAGAAATCAAACAGAGG-3' (antisense) for Bcl-2 (57°C, 215bp), 5'-CTCTGCCTCCTGGGTTCA-3' (sense)and 5'-AAAGATGGTCACGGTCTGC-3' (antisense) for Bax (52°C, 220bp), 5'-GCAGGTAGCCCAGGACA-3' (sense) and 5'-GTGGCAATCTTGGTGAAGTA-3' (antisense) for Bak1 (50°C, 273bp), 5'-ACGGATTTGGTCGTATTGGG-3' (sense) and 5'-TGGAAGATGGTGATGGGATT-3' (antisense) for GAPDH (53°C, 208bp). The parameter of thermocycler was set as: stage 1, 50°C for 2 min, 1 cycle; stage 2, 95°C for 10 min, 1 cycle; stage 3, 95°C for 15 s and 55°C for 1 min, 35 cycles. Melting curve analysis was used to monitor production of the appropriate PCR product. CT (cycle threshold) values were standardized to CT values of GAPDH. Samples obtained from three independent experiments were used for analysis of relative gene expression using the 2^-ΔΔCT^ method as previously described.[21] Each PCR was performed in triplicate with controls.

### Statistical Analysis

The *t* test procedure was used to compare the relative gene expression of qRT-PCR, OD value of MTT and ratio of apoptosis. The data were calculated as the percentage mean ± S.E.M. *P* values <0.05 were considered as significant.

## RESULTS

### Expression of Shh, Patched, Smo and Gli1 mRNA in pancreatic cancer cells

**Figure 1:**
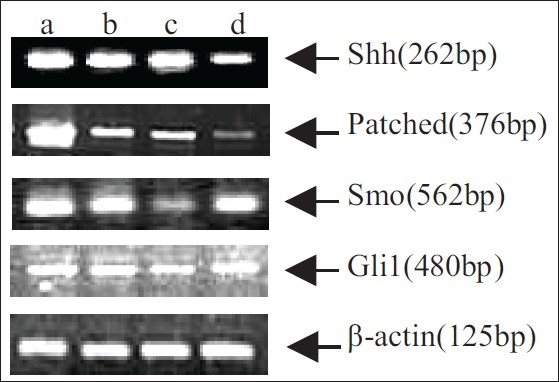
Expression of Shh, Patched, Smo and Gli1 mRNA in pancreatic cancer cells by using RT-PCR. Lane a: BxPC3 cell line; lane b: AsPC-1 cell line; lane c: Panc-1 cell line; lane d: SW1990 cell line

In order to determine whether Hh signaling pathway is activated in pancreatic cancer cell lines, RT-PCR assay was used to examine the expression of Shh, Patched, Smo and Gli1 mRNA in BxPC-3, AsPC-1, Panc-1 and SW1990 cells. The results showed that mRNA of Shh, Patched, Smo and Gli1 genes were expressed significantly [Figure 1]. Moreover, the IP-western assay was carried out as described in the Material and Methods section. As expected, the presences of Gli-1 proteins were detected in BxPC-3, AsPC-1, Panc-1 and SW1990 cells [Figure 5B].

### Inhibition of cell proliferation by cyclopamine or RNAi for Gli1

**Figure 2:**
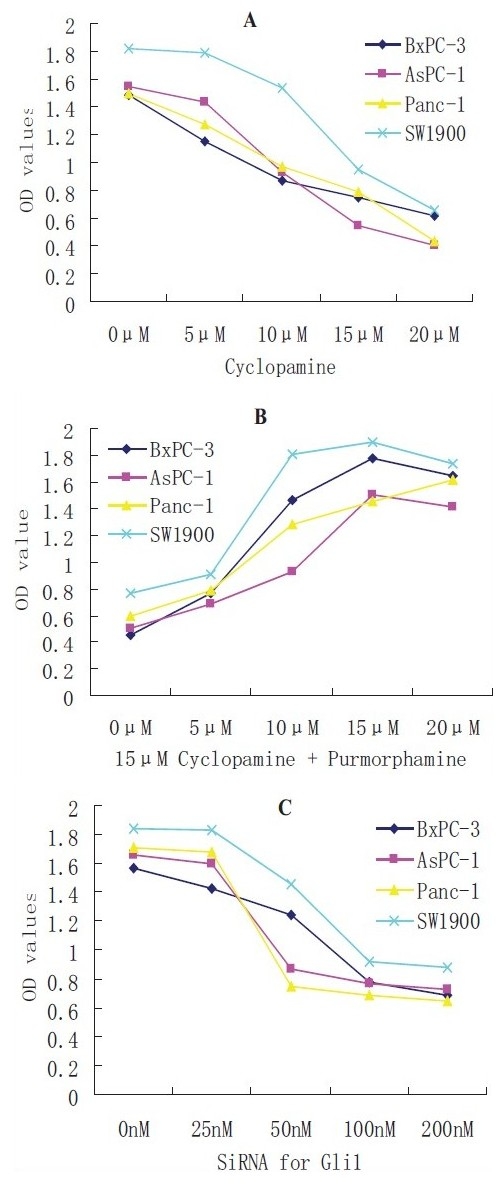
Proliferation of pancreatic cancer cell lines by MTT. A: proliferation inhibition of pancreatic cancer cells exposed to cyclopamine; B: purmorphamine reversed the proliferation inhibition of pancreatic cancer cells treated with 15 *μ*M cyclopamine; C: proliferation inhibition of pancreatic cancer cells by RNAi for Glil

The effects of cyclopamine or RNAi for Gli1 on cell proliferation were determined by the MTT assay. The results showed that after 4 days of exposure to cyclopamine, cell proliferation of BxPC3, AsPC-1, Panc-1 or SW1990 cells were suppressed in a dose-dependent manner that contrasted with the negative controls, and the IC50 concentration is about 15 *μ*M [Figure 2A]. Purmorphamine promoted proliferation of pancreatic cancer cells treated with 15 *μ*M cyclopamine in a dose-dependent manner and its efficacy peaked at about 15 *μ*M [Figure 2B]. For RNAi, MTT assay results showed that cell proliferations were suppressed dose-dependently by the duplexed siRNA oligonucleotides for Gli1 from 25 to 100 nM concentration, and there was no significant difference when the concentration was above 100 nM [Figure 2C].

### Apoptosis induced by cyclopamine or RNAi for Gli1

**Figure 3:**
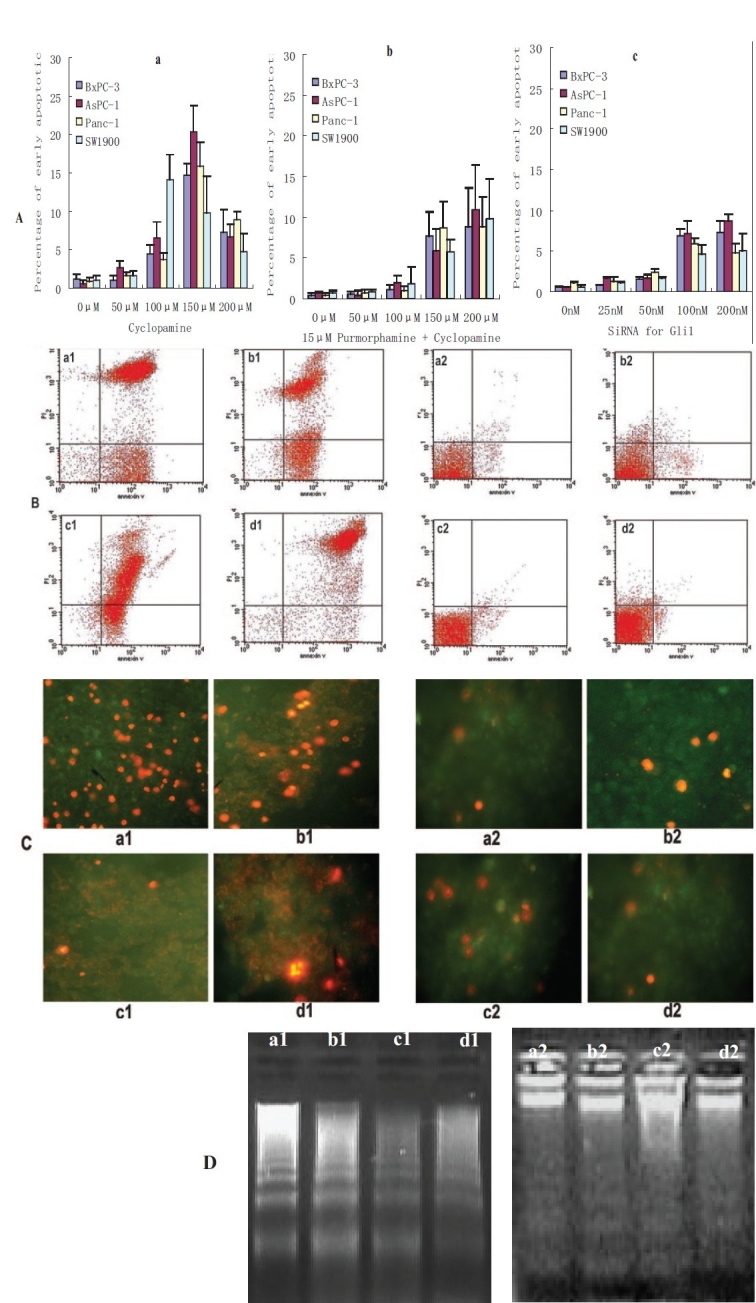
Apoptosis of pancreatic cancer cells. A: apoptosis percentage of pancreatic cancer cells. a: cyclopamine-induced apoptosis; b: purmorphamine counteract effect of cyclopamine; c: siRNA for Gli1. B: apoptotic cells exposed to 150 *μ*M cyclopamine or 100 nM siRNA for Gli1 by FACS assay. C: apoptotic cells stained by annexin V-FITC/PI and fluorescence microscopy; D: DNA ladder of apoptotic cells. (a1, b1, c1 and d1: pancreatic cancer cells exposed to cyclopamine; a2, b2, c2 and d2: pancreatic cancer cells exposed to siRNA for Gli1; a2: BxPC3; b1, b2: AsPC-1; c1, c2: Panc-1; d1, d2: sW1990.)

Annexin V-FITC/PI assay was performed to evaluate apoptosis of cultured pancreatic cancer cells induced by cyclopamine or RNAi for Gli1. FACS assay results showed that the apoptosis percentage of pancreatic cancer cells increased significantly when the concentration of cyclopamine was raised to 100 *μ*M (*p* < 0.05) and topped at 150 *μ*M cyclopamine (*p* < 0.01). The percentages of early apoptosis of pancreatic cancer cells treated with cyclopamine at the concentration of < 150 *μ*M were decreased significantly by 15 *μ*M purmorphamine (*P* < 0.05). For RNAi, the results showed that apoptosis percentage began to increase significantly until the dose was increased to 50 nM (*p* < 0.01), and increased dose-dependently from 50 to 200 nM; however, there were no significant difference between 100 and 200 nM (*p* > 0.05). [Figure 3A,3B]. After being exposed to 150 *μ*M cyclopamine or 100 nM siRNA for 24 h and treated with annexin V-FITC/PI, the pancreatic cancer was observed under fluorescence microscopy. The results showed most cells exposed to cyclopamine to have both annexin V-FITC and PI staining, and only small amount of cells were early apoptotic cells, indicated by the green fluorescence of Annexin V-FITC from cell membrane and devoid of PI staining. For RNAi there are a few cells displaying green fluorescence and several were PI stained. [Figure 3C].

### DNA fragmentation of pancreatic cancer cells exposed to cyclopamine or siRNA for Gli1

After being treated with 150 *μ*M cyclopamine for 24h DNA electrophoresis of BxPC-3, AsPC-1, Panc-1 and SW1990 cells showed the DNA ladders of apoptosis characteristic and some irregular DNA degradation indicated cell necrosis [Figure 3D]. For RNAi DNA electrophoresis showed there were no obvious DNA degradation [Figure 3D].

### Identification of Candidate Gli1 Binding Sites of Bcl-1 and IGFBP6 genes

**Figure 4:**
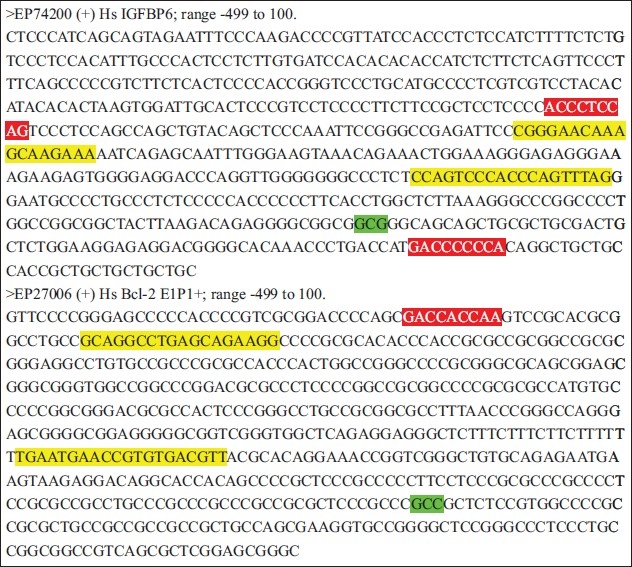
Gli1 binding sites and primers for XChIP-PCR. Green shadings are transcription initiation sites, red shadings are potential Gli1 binding sites and yellow shadings are primers for XChIP-PCP

In order to analyze relationship between transcription factor Gli1 and Bcl-2 or IGFBP6 genes, we searched potential Gli1 binding sites within the promoters and 5'-UTR regions of Bcl-2 (EP74200) and IGFBP6 (EP74200). Highly homologous sequences (the difference is no more than one base) were found in these promoters by homology sequence analysis. The results showed there were two potential sites in IGFBP6 promoter (5'-ACCCTCCAG-3' and 5'-GACCCCCCA-3') and one Gli1 binding site in Bcl-2 promoter (5'-GACCACCAA-3'). [Figure 4]. The homology of each site is 89%. The PCR primers were designed according to no more than 600 bases containing the Gli1-binding sites within the two promoter sequences, respectively.

### Binding of Gli1 to promoter regions of Bcl-2 and IGFBP6 genes in pancreatic cancer cells

**Figure 5:**
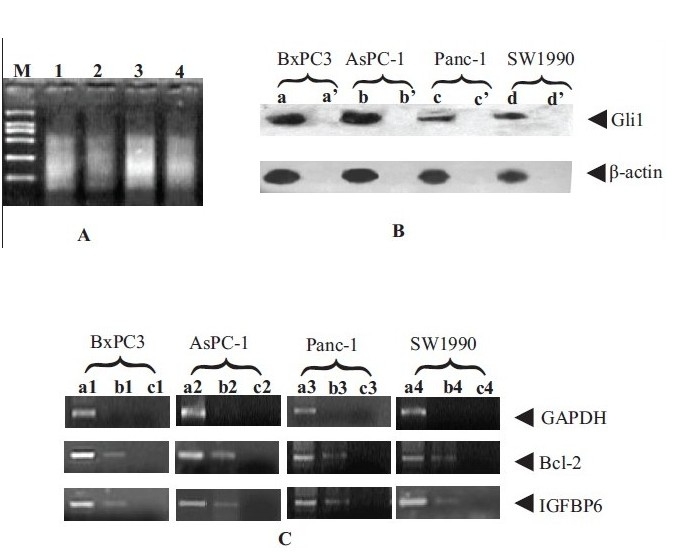
Binding of Gli1 to Bcl-2 and IGFBP6 genes by XChIP-PCR assay. A: total DNA sheared for 6 rounds of 10 s pulses (350 W, 60 s intervals). (M: BL200 DNA Marker; 1: BxPC3; 2: AsPC-1; 3: Panc-1; 4: SW1990.); B: a, b, c and d show expression of Gli1 protein compared with negative controls (a', b', c' and d') by IP-western assay; C: results of XChIP-PCR. the lanes of a1, a2, a3 and a4 were PCR products of positive control DNA templates; that of b1, b2, b3 and b4 were PCR products of DNA templates from Gli1 XChIP; that of c1, c2, c3 and c4 were negative controls

The results of chromatin sonication assay showed that the total DNA fragment sheared for six times was about 200–1000bp [Figure 5A]. The sheared samples were used as immunoprecipitation samples, and positive control templates of PCR. IP-western assay were carried out during XChIP assay as described in the Material and Methods section, and the results showed that Gli antibody used for assays could bind with Gli1 protein specifically under the same conditions [Figure 5B]. After that the XChIP-PCR assay was performed and the primer of promoter region of GAPDH was used as a negative control. The results showed that GAPDH, Bcl-2 and IGFBP6 were amplified from the positive contrasting template [Figure 5C], which confirmed the presence of GAPDH, Bcl-2 and IGFBP6 genes in the total chromatin fraction of pancreatic cancer cells. Thus, under the strict control, the negative amplification of GAPDH and positive amplification of Bcl-2 and IGFBP6 from DNA template recovered from immunoprecipitation compared with negative control provided evidence that transcription factor Gli1 bound to the promoters of Bcl-2 and IGFBP6 real time in the cultured pancreatic cancer cells [Figure 5B].

### Regulation of Shh, Gli1, IGFBP6, IGF2, PCNA, Bcl-2, Bax and Bak1 genes in pancreatic cancer cells by cyclopamine or RNAi for Gli1

**Figure 6:**
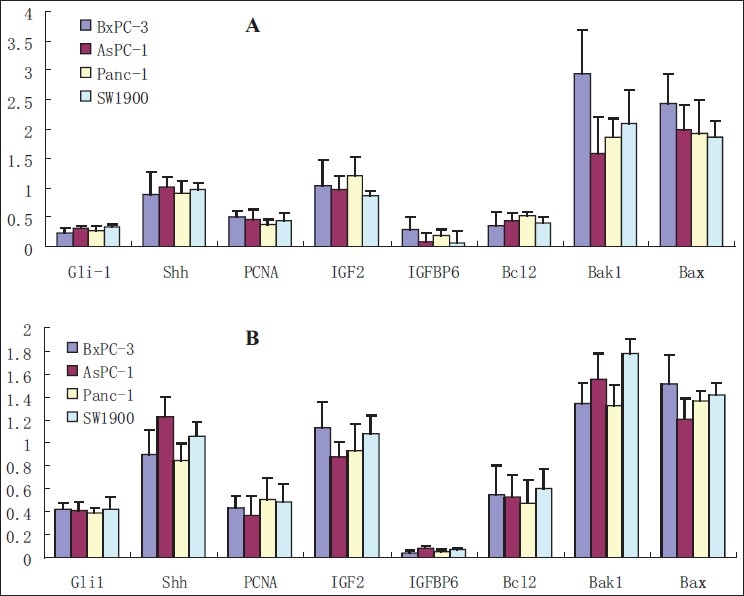
Expression analysis of relative genes mRNA by using QRT-PCR assay as described in the Materials and Methods section. A: Regulation of mRNA levels of Shh, Gli1, IGFBP6, IGF2, PCNA, Bcl-2, Bax and Bak1 genes by cyclopamine; B: Regulation of mRNA levels by RNAi for Gli1

Pancreatic cancer cell lines BxPC-3, AsPC-1, Panc-1 and SW1990 were treated with cyclopamine at final concentrations of 15 *μ*M or exposed to 100 nM duplexed siRNA oligonucleotides for 24 h. The results of qRT-PCR showed that regulation effects of 100 nM siRNA were similar to 15 *μ*M cyclopamine roughly. The main difference is that the inhibition to Gli1 and Bcl-2 were weaker and to IFGBP6 were stronger than that of cyclopamine. The relative mRNA levels of Gli1 were decreased to 0.23–0.34 folds in cyclopamine group (*p* < 0.01) and to 0.39–0.42 folds in RNAi group compared with negative control (*p* < 0.05). At the same time, relative expressions of IGFBP6, PCNA and Bcl-2 mRNA were decreased significantly (*p* < 0.05) and the levels of Bax and Bak1 mRNA were increased (*p* < 0.05) and Shh and IGF2 were not influenced (*p* > 0.05) [Figure 6].

### Expression level of Shh, Gli1, IGFBP6, IGF2, PCNA, Bcl-2, Bax and Bak1 mRNA in pancreatic cancer tissues

**Figure 7:**
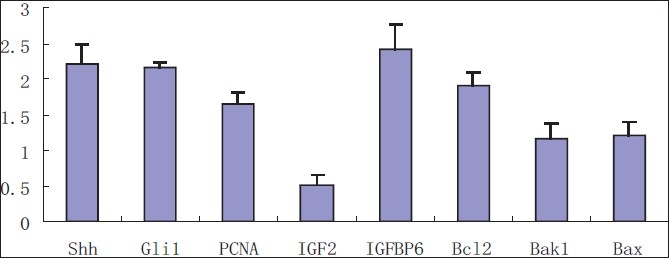
The result represented relative gene expression of Shh, Gli1, IGFBP6, IGF2, PCNA, Bcl-2, Bax and Bak1 mRNA of pancreatic cancer tissues (n = 6) compared with cancer side tissues (n = 6)

QRT-PCR was performed to evaluate the levels Shh, Gli1, IGFBP6, IGF2, PCNA, Bcl-2, Bax and Bak1 mRNA in pancreatic cancer tissues (*n* = 6) and corresponding cancer side tissues (*n* = 6). The results showed that relative mRNA levels of Shh, Gli1, IGFBP6, PCNA, Bcl-2 and Bax mRNA were increased with 2.21-fold, 2.16-fold, 1.65-fold, 2.43-fold, 1.9-fold and 1.21-fold, respectively (*p* < 0.05) and at the same time the expression of IGF2 mRNA was decreased to 0.52-fold (*p* < 0.05) in pancreatic cancer tissues compared with cancer side tissues. [Figure 7].

## DISCUSSION

Hh signaling pathway is one of the important signaling cascades during the development of insects and mammals.[22,23] This signaling drives cell proliferation in some cell types while causing differentiation in others during embryogenesis.[24,25] To date, multiple lines of evidence have proven that Hh signaling pathway is correlated with development of some types of cancers. Aberrant activation of Hh pathway in cancers is caused either by mutations of negative regulator in the pathway such as Patched in basal cell carcinomas or through ligands overexpression such as Shh in pancreatic cancer.[26] The downstream transcription factors, Gli1 2 and 3, bind to the same DNA sequence (5'-GACCACCCA-3').[27,28] Gli2 and 3 have both transactivation and repression domains, whereas Gli1 has been suggested to function only as a transactivator. Aberrant activating of Hh pathway leads transcription factor Gli1 into nucleus to bind the promoter region of target genes and promote gene transcriptions, which maintains the biologic behaviors of cancer cells.[29] Continuous activation of Shh-Gli1 signaling cascade has recently been implicated in the growth of a number of human malignancies.[30,31] In our study, we validated reactivation of Shh-Gli1 signaling pathway in pancreatic cancer tissues and cultured cancer cells by testing the main components of Shh-Gli1 signaling pathway using qRT-PCR or IP-western.

To study its relationship with cell survival, we investigated proliferation and apoptosis of cultured pancreatic cancer cells by inhibiting the activity of this signaling pathway using cyclopamine, a specific small molecule inhibitor of Smo protein. The results showed that cyclopamine inhibited cell proliferation and induced apoptosis *in vitro*. However, in our experiments, cell proliferation was suppressed dose dependently at low concentration of cyclopamine, but apoptosis increased significantly until concentration of cyclopamine increased to about 10 times of IC50 of proliferation inhibition compared with negative control. To determine whether cyclopamine has unspecific off-target effects, we used purmorphamine to counteract the efficacy of cyclopamine to pancreatic cancer cells. Purmorphamine is a specific small molecular agonist of Smoothened protein and can activate the Hh pathway of cells treated with cyclopamine.[32] The results showed that purmorphamine can reverse the proliferation inhibition effect of cyclopamine completely at low concentration and counteract apoptosis-inducing effect of higher concentration cyclopamine partly. Therefore, it is logical to conclude that the influence of cyclopamine to cell survival at low concentration is mainly due to proliferation inhibition, and at high concentration we could not exclude nonspecific effect or cytotoxic effect. Recently it has been reported that cell lines grown *in vitro* may lose their dependence on Hh pathway for survival and that the high concentrations of cyclopamine required inhibiting cell growth, may be due to nonspecific effects.[33,34]

In order to confirm the relationship of Hh-pathway with cell survival, we carried out RNAi assay for Gli1. The results showed that the duplexed siRNA suppressed cell proliferation and induced apoptosis. However, the apoptosis percentage was lower than that of cyclopamine significantly, and there was no obvious necrosis. This suggests that the Hh signaling pathway maintains cell survival mainly by promoting proliferation though it is involved in apoptosis.

Although a large body of evidence shows that Hh signaling is related to development of cancer, the mechanism is unclear yet. Since the Shh-Gli1 signaling pathway is reactivated in pancreatic cancer, the mechanisms of Hh signaling promoting development of cancer will undoubtedly, to a great extent, depend on target genes of transcription factor Gli1.[29] It is a common distinctive characteristic of all Gli1 targets that there are highly homologous sequences to Gli1 binding site. We can figure out Gli1 targets initially through analyzing the promoter sequences of genes finding out the Gli1 DNA-binding consensus site. Hallikas, *et al* have carried out genome-wide prediction of mammalian enhancer for Gli1 through computational tool and revealed the presence of multiple tissue-specific enhancers in mouse c-Myc and N-Myc genes.[35] This suggests that the target genes of Gli1 could be found out through this analogical method in cancer. Consequently, we analyzed the promoter and 5'-UTR sequences of Bcl-2 and IGFBP6 and found some highly homologous sequences with Gli1 binding element. A potential site, 5'-GACCACCAA-3', was found in E1P1 + promoter of Bcl-2 (EP27006), which has recently been demonstrated to be responsible for the transcriptional regulation of Bcl-2 through Hh signaling pathway.[36] For IGFBP6, two Gli1 binding sites, 5'-ACCCTCCAG-3' and 5'-GACCCCCCA-3', were revealed in its promoter regions (EP74200). In this study, we demonstrated that Gli1 protein bound to the promoters of IGFBP6 and Bcl-2 by XChIP-PCR assays, which suggested that Gli1 facilitates transcriptions of IGFBP6 and Bcl-2 in a parallel manner in pancreatic cancer cells.

The expression of Gli1 mRNA was down-regulated significantly by cyclopamine or siRNA for Gli1 demonstrated that cyclopamine or siRNA for Gli1 inhibited the activation of Hh signaling partly. Thus, PCNA mRNA decreasing suggests that Hh signaling promotes proliferation of pancreatic cancer cells. IGFBP6 is a Gli1 target as reported previously[37] and usually binds with IGF2 with high affinity and prevents IGF2-mediated effects. However, over expressed IGFBP6 may be involved in maintaining the proliferative state of cells and preventing cellular differentiation in several tumors.[38,39] Our results showed that mRNA of IGFBP6 was highly expressed in pancreatic cancer tissues and down-regulated significantly in cultured pancreatic cancer cells by cyclopamine or RNAi for Gli1. On the other hand, the expression of IGF2 was very low in pancreatic cancer and was not affected by cyclopamine or RNAi for Gli1, which means the regulation of IGF2 expression is independent of Shh-Gli1 signaling pathway in pancreatic cancer.

Thus, it seems that the overexpressed IGFBP-6 resulting from Gli1 expression was independent of IGF2. However, IGF2 has been reported to be an important islet cell hormone in endocrine islet cells, which are dispersed throughout the exocrine pancreas that could act via a proxicrine mechanism on pancreatic cancer cells,[40,41] so it could not be excluded soundly that the upregulation of IGFBP6 by Gli1 may only be a response of pancreatic cancer cells to the stimulus of paracrine IGF2 from islet cells.

Bcl-2 family included the pro-apoptotic genes (such as Bak1, Bax) and anti-apoptotic genes (such as Bcl-2, Bcl-X). Bcl-2 protein forms heterodimers by binding to the Bax and Bak1 protein and protects cell from apoptosis.[16,42] In this study, the Bcl-2 mRNA was down-regulated as well as Bax and Bak1 up-regulated in pancreatic cancer cells by cyclopamine or RNAi for Gli1. This suggested that Hh signaling suppressed the apoptosis though regulating not only the transcription of Bcl-2 directly but that of Bax and Bak1 by some unknown mechanisms as well. Although, cyclopamine changed expression of apoptosis-related genes at low concentration, there were no apoptotic characteristics until cyclopamine reached high concentration. These results suggest that apoptosis might be regulated by multiple mutual restrictive factors, and apoptosis is present only when the balance among these factors is broken.

Overall, these data suggested a model in which Shh-Gli1 signaling controlled surviving state of pancreatic cancer cells by regulating Bcl-2 and IGF family in a parallel manner. On one hand, Shh-Gli1 signaling acts to inhibit apoptosis of pancreatic cancer cell by directly activating expression of Bcl-2 and indirectly decreasing the levels of Bax and Bak1. On the other hand, Shh can directly stimulate expression of IGFBP6 through Gli1, which is reported to be involved in maintaining the proliferative state of cells and preventing cellular differentiation.

In conclusion, Gli1 directly facilitates expressions of IGFBP6 and Bcl-2 at the same time in cultured pancreatic cancer cells. Moreover, upregulation of IGFBP6 and Bcl-2 following the reactivation of Shh-Gli1 pathway in pancreatic cancer tissues suggest regulation model of Shh-Gli1 pathway to IGFBP6 and Bcl-2 is similar *in vivo*. This might be one of the mechanisms through which Hh signaling pathway maintains cell survival and promotes development of pancreatic cancer, which imply that Shh-Gli1 pathway might be an effective target of treatment for pancreatic cancer. Our study contains an innovative intriguing idea that by searching potential Gli1 binding sites in promoter sequences followed by XChIP assay, we could identify novel Hh target genes that might provide insights in the mechanism of Hh-related cancer development.

## AUTHOR'S PROFILE

**Dr. Xuan-Fu Xu, M.D.**

2008 - : Assistant Professor, Doctor in Charge, Department of Gastroenterology, the tenth hospital, Tongji University, China.

Specialty and Present Interest: Development and Cancer; Biomarkers of carcinogenesis and cancer chemoprevention
